# Subtleties in Bayesian decision-theoretic analysis for forensic findings: Notes on recent discussion of the role of validation study data in rational decision making

**DOI:** 10.1016/j.fsisyn.2024.100548

**Published:** 2024-08-31

**Authors:** Alex Biedermann

**Affiliations:** University of Lausanne, Faculty of Law, Criminal Justice and Public Administration, School of Criminal Justice, 1015, Lausanne–Dorigny, Switzerland

**Keywords:** Bayesian decision theory, Error rates, Feature agnosticism, Inconclusive, Relevance, Probability

## Abstract

This technical note extends a recent discussion in this journal of the role of validation study data in rational decision making. One argument that has been made in this context, using elements of Bayesian decision theory, is that further aggregation of validation study data into error rates involves a loss of information that compromises rational inference and decision making and should therefore be discouraged. This technical note seeks to explain that this argument can be developed at different levels of detail, depending on the definition of the propositions of interest, the forensic findings to be evaluated (and hence the form of the likelihood ratio), and the characterization of the relative desirability of decision consequences. The analyses proposed here reveal the cascade of abstractions and assumptions into which discussions about the use of validation study results in forensic science have fallen. This reinforces the conclusion that further aggregation of validation study data into error rates is problematic. It also suggests that even if a definition of error rate(s) could be agreed upon and defensively quantified in a given application, we should rethink and possibly adjust our expectations about what exactly error rates can practically contribute to rational modes of reasoning and decision making in legal contexts.

## 1 Introduction

A recent article in *Forensic Science International: Synergy* by Swofford et al. [27] provides a valuable overview of the intense debate over how to deal with the examiner response category “inconclusive” in the context of error rate-based performance evaluation of forensic examiners and the methods they use. In Appendix I of their paper, the authors reproduce elements of the Bayesian decision theory (BDT) account of factfinding. The authors argue that “(…) likelihood ratios, rather than error rates, are the quantities of interest (…) for factfinders” [27, p. 8]. The authors further assert that “(…) it is critical that factfinders have access to information that would assist their assessments of these [likelihood] ratios. Summarizing performance using error rates alone (…) deprives the factfinder of information relevant for updating their beliefs” [27, p. 10]. More specifically, Swofford et al. [27] argue that data from validation studies, which are typically presented in e.g. 2 × 3 tables,^1^ should *not* be further summarized, contrary to what is advocated by some proponents of further aggregation of data to error rates. This processing of the data, of which there are several approaches, must somehow deal with the response category “inconclusive”. This aspect is at the heart of the current controversy on how to handle data from validation studies, since different procedures^2^ for handling the response category “inconclusive” can lead to differences in the resulting error rates. Swofford et al. [27] argue that a coherent decision maker requires a complete view of how the results are distributed across the different examiner conclusion categories.

While the position of Swofford et al. [27] follows broadly from their formulaic development, their intention was to limit the scope of their development to illustrate the main point that rational decision makers can benefit from having access to a full 2 × 3 table. However, there are further levels of complication to this argument, and it is worth exploring some of those additional levels. The purpose of this technical note is to provide constructive comments on several related aspects, namely the definition of the propositions of interest, the forensic findings to be evaluated (and hence the form of the likelihood ratio), and the characterization of the relative desirability of decision consequences. These aspects – when considered in the Bayesian decision-theoretic formalization – provide additional insight into the inferential challenges posed by scientific evidence beyond the focused topic of how to use data from validation studies and their summaries.

## 2 On propositions

Swofford et al. [27] begin their analysis by defining “(…) the prosecution hypothesis *H*_*p*_ that the two impressions share the same source, relative to the defense hypothesis *H*_*d*_ that they do not” [p. 8]. This is commonly referred to as a pair of source-level propositions [11]. Next, they make the assumption “(…) that the factfinder has only two actions available–find the defendant “guilty” or find the defendant “not guilty.”” [p. 8] The core of the authors’ development is the calculation of the expected costs of each of these two decisions in light of a forensic examiner’s report of an identification.

It is worth pausing for a moment to reflect on the choice of source-level propositions. While such propositions may seem close to the nature of the expert’s report on the results of comparative examinations, and thus a practical choice for the purpose of presenting an example based on minimal considerations, such propositions are far removed from the ultimate decisions with which the factfinder is concerned. In reality, we would hope that factfinders would base their ultimate decision (verdict) *not* (or at least not directly) on their degree of belief in source-level propositions, but on crime-level (also called offense-level [11]) propositions of the type “the person of interest (POI) is the perpetrator” vs. “an unknown person is the perpetrator”, conditioned not only on the forensic evidence, but on *all* the evidence that bears on the ultimate issue.

To be clear, BDT is a liberal concept and does not tell us which states of nature (here propositions) to choose to define decision consequences (outcomes), and Swofford et al. [27] are free to focus on source-level propositions. In fact, BDT merely tells us how to combine probabilities (for propositions) with utilities or losses (or “costs” in the case of Swofford et al.) *when* propositions are chosen in a certain way, i.e., how to compute expected values (here: costs) for characterizing and comparing rival decisions. However, it is worth asking whether conditioning on source-level propositions is the most sensible modeling choice for the problem under consideration here. Put another way, the question is how to relate aspects of a formal development to aspects of the real-world problem of interest. As Professor Kaye has noted, “no mathematical result is self-applying, and additional argument is necessary to bridge the gap from a general mathematical truth to a substantive application – in law as in any other domain” [18, p. 27]. Fortunately, there is a way to show that, under certain assumptions, an account based on source-level propositions leads to the same results in terms of expected costs as an account based on offense-level propositions.

Before explaining this, however, it is worth anticipating a possible objection. Forensic practitioners sometimes claim that offense-level propositions are beyond the scope of the forensic scientist’s work. This is a truism, but it does not mean that offense-level propositions could not be used to define likelihood ratios, because forensic scientists should not comment directly on propositions, regardless of their hierarchical level. Instead, forensic examiners should assess the probability of their findings *given* propositions and conditioning, task-relevant information [e.g. 30]. Therefore, there is no problem with propositions being at the crime-level, as long as the forensic scientist focuses on the findings given the propositions and not the reverse. However, there is a practical obstacle to this because, as discussed below, evaluating the findings given higher-level propositions requires additional assessments beyond the rarity of the analytical features. These additional assessments depend on the circumstances of the case, and forensic scientists may not feel competent to incorporate these assessments into their evaluation, which may explain their reluctance to evaluate their findings with respect to offense-level propositions.

Returning to Swofford et al.’s [27] development, there are at least two ways to resolve the argumentative gap between a factfinder’s decisions and source-level propositions. One way, the simpler one, is to change the definition of decisions but leave the definition of propositions unchanged. Another way, the more extensive one, is to do the opposite: leave the definition of decisions unchanged, but extend the model to include additional propositions. These two options are discussed in the following sections. Box 1 provides a more detailed explanation using graphical models (influence diagrams).

Consider the first option. It consists in redefining the decisions “convict” and “acquit” as “(*decide* to) consider the compared items as coming from the same source” and “(*decide* to) consider the compared items as coming from different sources”.^3^ This is a natural way to define decisions when the uncertain states of nature under consideration relate to the source of the items being compared. Such a modified development amounts to the decision-theoretic account of identification [6,7], which may be an appropriate perspective for a legal decision maker, but not for a forensic examiner [10]. In summary, the option of changing the definition of decisions (actions) leaves the formulaic development of Swofford et al. [27] unchanged. What changes is the *meaning* given to some of the components of the formulaic development: the definition of decisions as verdicts is downgraded to an inferentially more modest level, i.e. source attribution.^4^ The latter, source attribution, seems more appropriate because the former, a decision on ultimate issues (verdict), requires more than a stance on source-level propositions. The additional elements required become apparent in the second option, which is explained below.

In the second option of modifying Swofford et al.’s [27] development, the “convict” and “acquit” decisions remain unchanged, but the propositions are extended from the source-level to the crime-level. As noted above, this change seems necessary because we expect legal decision makers to base their verdict on their view of the proposition whether the person of interest is the perpetrator or not, based on all the information and evidence available in the case, rather than just source-level propositions regarding a particular piece of forensic evidence. This is not simply a semantic distinction, as the likelihood ratio – the role of which is rightly emphasized by Swofford et al. [27] – involves several additional considerations, in particular relevance and the probability that the recovered trace was left for innocent reasons [14]. Moreover, for some types of traces, especially those left by shoe soles or tools, there is an additional source of uncertainty, i.e., whether the person of interest used the particular shoe or tool in the event that he or she is indeed the perpetrator and the trace was left by the perpetrator. This leads to an additional variable in the formulaic development of the likelihood ratio [15].

As an example of a likelihood ratio with crime-level propositions, consider a hypothetical case, adapted from [30, p. 12], involving a single trace (a large fresh bloodstain) found at the scene of a crime committed by a single perpetrator.^5^ The person of interest says that she has never been on the premises. Denote this information by *I*. The finding to be evaluated, *E*, is that the DNA profile of the POI corresponds to the DNA profile of the bloodstain. Let the propositions of interest be “The POI is the perpetrator” (*H*_*p*_) and “An unknown person is the perpetrator” (*H*_*p*_). For such a situation, the likelihood ratio V = Pr(*E*∣*H*_*p*_, *I*)/Pr(*E*∣*H*_*d*_, *I*) can be shown to correspond to Ref. [28]:

(1) $$\text{V}=\frac{r+\left(1-r\right)\gamma }{r\gamma +\left(1-r\right)\left[p+\left(1-p\right)\gamma \right]}$$

where *p* is the probability that the POI left the trace for innocent reasons, *γ* is the proportion of the population of interest that has the analytical characteristics of the trace, and *r* is the probability of relevance (i.e., the probability that the bloodstain was actually left by the perpetrator). Eq. (1), the likelihood ratio with crime-level propositions, reduces to 1/*γ* for cases where *r* = 1 (i.e., it is certain that the bloodstain was left by the perpetrator). But 1/*γ* is just the likelihood ratio for source-level propositions [e.g., 1]. Thus, Swofford et al.’s [27] calculation of expected costs for decisions about ultimate issues based on source-level propositions leads to the same result as calculating expected costs for the *same* decisions but based on crime-level propositions, *assuming that* the relevance of the crime stain is undisputed, which justifies Swofford et al.’s [27] intention to focus on a narrow example. The same result can be obtained for a type of trace (evidence) more closely related to the discussion in Swofford et al. [27], such as toolmarks, but with some additional assumptions as discussed in Ref. [15].

One might object that the above development, especially the likelihood ratio 1/*γ*, does not exactly reflect the analysis of Swofford et al.’s [27], because the finding they focus on is not an actual correspondence between features, but a scientist’s *report* of a source attribution conclusion. This is true with respect to the definition of the findings, but makes no difference from a structural point of view. If *E* is a feature correspondence, then the probability of *E* given the proposition that the compared items come from different sources is *γ*, which expresses the rarity of the features. If *E* is defined as a scientist’s report of a source attribution (identification), then the probability of *E* given the different source proposition is, in Swofford et al.’s [27] notation, Q1, the probability of a false positive report.

In summary, the above discussion of Swofford et al.’s [27] analysis shows that their insistence on the importance of the likelihood ratio as a component of rational decision making at advanced stages of the legal process is a valid point from a formal analytical perspective. It should be noted, however, that their development is based on a reduced set of considerations, making it a special case of a broader account that distinguishes between decisions about ultimate issues based on crime-level propositions and decisions based on source-level propositions. Moreover, with crime-level propositions as the inferential target, the probability of a false positive result – and thus data suitable for assessing such a probability (e.g., from validation studies) – is a *necessary but not sufficient* consideration. Other aspects, such as the relevance of the trace or mark of interest (i.e., whether it was left by the perpetrator) play an essential role. In other words, it is as important to insist on the role of the likelihood ratio in coherent decision making under uncertainty as it is to be precise about the analytical form of the likelihood ratio, especially the definition of the target propositions. Likelihood ratios can differ because of differences in the definitions of their components, but these different likelihood ratios can, under certain assumptions, lead to equivalent overall conclusions when calculating the expected costs (or: utilities, losses) of decisions.

Box 1 **Graphical representation of variations in decision-theoretic modeling**.

**Fig. 1:**
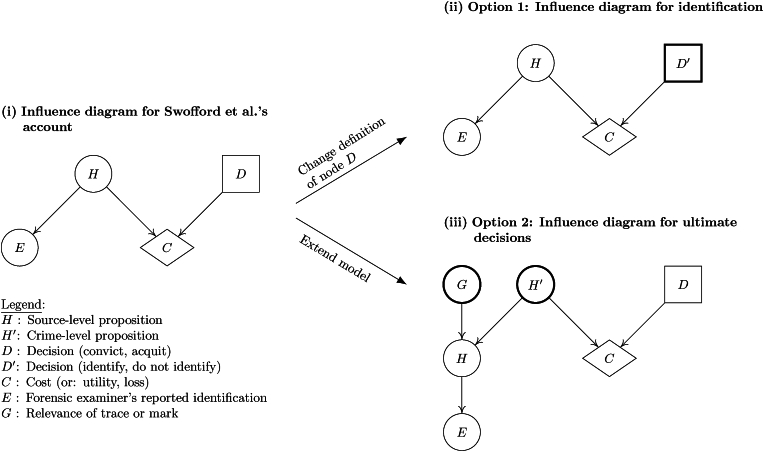
Influence diagrams (Bayesian decision networks) for (i) Swofford et al.’s [27] account of decision making about ultimate issues (based on source-level propositions), (ii) identification (source attribution), and (iii) decision making about ultimate issues (based on crime-level propositions). Circled nodes represent probabilistic variables (propositions), square nodes represent decisions, and diamond-shaped nodes represent the cost (or: utility, loss) function. Bold bordered nodes in models (ii) and (iii) highlight differences with respect to model (i). For simplicity, informational links between findings nodes (*E*) and decision nodes (*D*, *D*′) are omitted. All probabilistic and decision nodes are binary.

As noted in the body of the text, Swofford et al.’s [27] account focuses on decisions about ultimate issues and the computation of expected costs for such decisions under source-level propositions, conditioned on the forensic examiner’s report of an identification. This view can be summarized by the influence diagram shown in Fig. 1(i).

To avoid the conceptual gap in this model between ultimate issues and source-level propositions, Fig. 1(ii) uses a slight modification of the definition of the decision node: i.e., instead of defining the decisions as “convict” and “acquit” (node *D*), the node *D*′ is defined as “identify” and “do not identify” *from the factfinder*’*s point of view*. The model (ii) defines the computation of the expected cost (or: utility, loss) of the decision to consider the compared items as coming from the same source (or from different sources), *given* the forensic examiner’s report of an identification, represented by the node *E* (for more details see also [6,28]).

If one wants to maintain the definition of the decisions “convict” and “acquit”, then propositions for calculating expected costs (or: utilities, losses) should be defined at the crime-level [8]. However, this requires constructing an argument from source-level to crime-level propositions that includes considerations of the relevance of the examined trace or mark (i.e., whether it was left by the perpetrator or not). A way to achieve this extension formulaically has been described in Ref. [14]. A graphical model (Bayesian network) for this development has been provided in Ref. [16] and is reproduced here in Fig. 1(iii) in terms of nodes *H*, *H*′, *G* and *E*.

Model (i) can be seen as a special case of model (iii) for situations where there is no uncertainty as to whether the examined trace or mark comes from the perpetrator or not. In such situations, it can be shown that the two models lead to equivalent results in terms of expected cost (or: utility, loss) for ultimate decisions (*D*), because the likelihood ratio for source-level propositions, Pr(*E*∣*H*_*p*_, *I*)/Pr(*E*∣*H*_*d*_, *I*), is equivalent to the likelihood ratio for crime-level propositions, $\mathrm{Pr}(E|H_{p}^{\prime },I)/\mathrm{Pr}(E|H_{d}^{\prime },I)$.

## 3 Feature agnosticism in defining the results to be evaluated

In addition to propositions, it is also worth considering the definition of the findings to be evaluated. In the development of Swofford et al. [27], a broad perspective was chosen in which the findings could take any form in which the expert may express his or her conclusions. In Swofford et al.’s [27] example, the expert reports an identification, and it is to this expert *utterance* that the recipient of expert information assigns an expression of probative value (in the discussion here, a likelihood ratio). It is important to understand the implications of this perspective.

In particular, it is important to understand that assigning a likelihood ratio to the expert’s *utterance*, i.e., the identification report, rather than to the actual observed characteristics (features) of the trace or mark under examination, leads to *feature agnosticism*. That is, the selectivity of the features observed in the case at hand, e.g. the rarity of a DNA profile in the case of biological traces, is no longer part of the considerations [5]. Instead, attention is reduced to the sole general performance of the expert and/or method as measured in the validation study.^6^ Moreover, the choice of defining the findings in terms of the expert’s report of an identification is problematic because it could be interpreted as suggesting that it is acceptable for experts to express themselves in terms of “identification” and other conclusions that amount to a statement about whether or not the items being compared came from the same source. To be fair, this is not the fault or intent of Swofford et al. [27], as their choice of definition for the findings is a mere consequence of their laudable engagement with the currently most common forensic reporting format [25], as well as with the results of the now fashionable black-box studies. In addition, the first author in Ref. [27] is well known for having demonstrated the feasibility of developing and implementing alternative assessment and reporting formats in previous work [e.g., 23,24,26].

While defining the findings to be evaluated in terms of an expert’s reported identification captures one of the most common reporting formats, it is by no means a necessary condition. In fact, it is possible to provide likelihood ratios for source-level, and by extension crime-level, propositions based on an examiner’s *report of an observed correspondence* (of features) rather than a reported identification. An example is the likelihood ratio formula described by Thompson et al. [29]. Again, this may seem to be a pedantic emphasis on a purely semantic distinction. However, the distinction is paradigmatic [22] and worth repeating. The likelihood ratio described by Thompson et al. [29] distinguishes between an actual correspondence between the features of two items being compared on the one hand, and a scientist’s *report* of an observed correspondence, on the other. Their development shows how the rarity of the corresponding features (informally referred to as the “random match probability”) and the “false positive probability” [29, p. 49], i.e., the probability that the examiner reports a correspondence when in fact the compared items do not have corresponding features, affect the value of the likelihood ratio. It is important to understand the conceptual implications of this approach with respect to accounts that are agnostic about the actual features.

In fact, when the information to be evaluated is the expert’s report of an identification, regardless of the selectivity (i.e., rarity) of the features observed by the forensic examiner, this can lead to an over- or undervaluation with respect to those features. The reason for this is that the finding is reduced to a non-specific utterance of “identification”, the value of which is characterized by some sort of aggregate performance measure that is largely disconnected from the specifics of the case at hand. Although one may argue that care is taken to ensure that the present case falls within the so-called “general notion of range of validation” [17, p. 832] from which performance measures have been derived, this does not resolve the fundamental disconnection from the features observed on the items examined in the case at hand.

In contrast, the likelihood ratio of Thompson et al. [29] takes into account both the rarity of the features actually observed by the examiner *and* the probability of a false positive report. The latter is not a mere aggregate measure of performance (e.g., error rate), but a case-specific assessment of the probability that the examiner would report corresponding features *in the given case*, even though the compared items do not have corresponding features. This assessment may well vary depending on the quality and quantity of the items examined, the conditions under which they were collected, stored and examined, the skill of the examiner, etc. Overall, such a likelihood ratio should result in a more case-tailored assessment.

One might object to the above on the grounds that likelihood ratios that reduce to ratios of, for example, the probability of the examiner reporting “identification” when *H*_*p*_ is true and the probability of the examiner reporting “identification” when *H*_*d*_ is true are merely a result of the nature of the available data (typically from black box studies) and a way of making the best use of such data. Such an argument, however, would amount to putting the cart before the horse: it would amount to defining likelihood ratios to “fit” the available data, rather than first thinking about the most appropriate likelihood ratio format and then finding the appropriate data to quantify it. The former approach tends to perpetuate the outdated identification paradigm and reduce attention to black box testing, where much of current forensic science research and practice remains stuck. While it is true that for many non-DNA disciplines where features are difficult to measure, this approach remains – for practical purposes – a primary choice, this limitation should not be taken to mean that other, more aspirational, perspectives are not available.

The calculation of expected costs according to the models discussed in Box 1 is compatible with this perspective. In fact, it is possible to modify the influence diagram shown in Fig. 1(iii) by replacing the node *E*, defined as the examiner’s *reported identification*, with the network fragment *F* → *C*, where *F* represents the proposition that the compared items have corresponding features and *C* represents the proposition that the examiner reports the observation of corresponding features, and *F* receives an incoming arc from the source-level proposition *H* (see also [28] for more technical details). The resulting model allows one to compute expected costs based on probabilities for crime-level propositions obtained by a coherent combination of Thompson et al.’s [29] source-level likelihood ratio and Evett’s [14] likelihood ratio for crime-level propositions.

## 4 On the cost ratio

As part of their development, Swofford et al. [27] are concerned with the ratio of the costs associated with the two adverse consequences that a decision about ultimate issues may lead to. Specifically, they define *C*_*wc*_ as the cost associated with the consequence of deciding to convict when in fact *H*_*d*_ is true. In turn, they define *C*_*fa*_ as the cost associated with the consequence of deciding to acquit when in fact *H*_*p*_ is true. The ratio *C* = *C*_*wc*_/*C*_*fa*_ and its comparison with the (posterior) odds^7^ of the propositions of interest is a well-known decision criterion in BDT [e.g., 4].

One question of interest here is what the ratio *C* means. Swofford et al. interpret it as follows: “The quantity *C* represents how many false acquittals the factfinder would exchange to avoid one false conviction” [27, p. 9]. This description, which is common in the literature, has a frequentist tone because it refers to the plural “acquittals”. However, the application of the BDT criterion is a case-specific task. The component costs *C*_*wc*_ and *C*_*fa*_ refer to the consequences of the decision *in the particular case*. This directs us to think about the stakes involved in the instant decision, i.e., the relative losses faced by the decision maker in the case at hand, rather than a conviction-error rate across many different cases. That is, the ratio *C* = *C*_*wc*_/*C*_*fa*_ expresses how many times worse the outcome *wc* (wrongful conviction) is, *in the case at hand*, compared to the decision outcome *fa* (false acquittal) from the decision maker’s perspective.^8^

## 5 Conclusions: a cascade of and assumptions

The question remains as to where exactly BDT arguments for decision making based on forensic findings, as presented in the forensic and legal literature, lead us. On the one hand, as Swofford et al. [27] show us, such developments have value in that they provide formal arguments for pointing out the limitations of using error rates resulting from further aggregation of forensic method and examiner performance data summarized in 2 × *k* tables (where *k* is the number of conclusion categories) at advanced stages of legal decision making. To some extent, however, this conclusion is not surprising, as it results from evaluating a descriptive concept (i.e., a summary statistic with respect to the *aggregate case*) against the properties and requirements of a rich inferential concept, here BDT, which combines probability and utility theory to deal with *case-specific decision making*. On the other hand, the conclusion that error rates *alone* are inadequate, and that instead likelihood ratios are relevant quantities for fact-finders, does not solve the challenges posed by forensic science evidence, for a number of reasons.

First, likelihood ratios come in a variety of forms, depending on the definition of the findings being evaluated and the competing propositions of interest. The most commonly produced likelihood ratios – if they are produced at all – relate to propositions about the source (origin) of evidential material. However, such propositions are far removed from the higher-level propositions involved in the decision making of the recipients of expert evidence. Although it is theoretically possible to extend source-level likelihood ratios to higher-level propositions, such extensions involve considerations, such as relevance, that are beyond the expertise of the forensic examiner. Moreover, such formal developments quickly reach levels of complexity that make them inaccessible to real-world decision makers. Second, from a practical point of view, discussions based on formal reasoning methods, such as those considered here, remain theoretical or aspirational at best, to the extent that the understanding of the nature and actual mode of functioning of trials are predicated on other perspectives [e.g., 2]. There are connections between these perspectives and formal methods of reasoning [e.g., 3,12], but these considerations have yet to attract the attention of forensic science researchers and practitioners.

Taken together, the above considerations reveal the cascade of abstractions and assumptions into which discussions about the use of validation study results in forensic science have fallen. This leads to an impasse in the sense that even if the field could overcome the sheer endless ways in which attempts to establish error rates can be attacked, the concept of error rate itself remains elusive [13]. Its role, at least in formal accounts of reasoning under uncertainty and in *coherent decisionalism* [19], is very narrowly defined: it can *aid* inference, but not replace it, let alone anticipate a decision. This does not necessarily diminish the importance of error rates and debates surrounding them, but it does suggest that we should rethink and possibly adjust our expectations about what exactly error rates can practically contribute to rational modes of reasoning and decision making in legal contexts.

## CRediT authorship contribution statement

**Alex Biedermann:** Writing – review & editing, Writing – original draft, Funding acquisition, Formal analysis, Conceptualization.

## Declaration of competing interest

The author declares that he has no known competing financial interests or personal relationships that could have appeared to influence the work reported in this paper.

## Footnotes

1 For example, a 2 × 3 table results from a study in which the ground truth is binary (i.e., the examined items come from the same source or from two different sources) and there are three conclusion categories, such as “identification”, “inconclusive” and “exclusion”. Note that the range of conclusion categories is not limited to three levels. As Swofford et al. [27] explain, one could also use “a 5-level scale, 7-level scale, 9-level scale, or another similar type of scale” [27, p. 9].

2 For example, some procedures ignore the conclusion category “inconclusive” while others combine it with other categories such as “identification” or “exclusion”.

3 Put another way, the decision here amounts to accepting (not accepting) the source-level proposition, i.e. making a source attribution (identification) decision.

4 Note that, strictly speaking, this model moves away from the ultimate decision. The change in the definition of the decision variable is only intended to align decisions and propositions at the source level.

5 For an extension to *k* perpetrators, see Ref. [14]. For an extension to mark evidence, see Ref. [15].

6 This can lead to additional problematic practices, such as the computation of the positive predicitive value (PPV) as a proposal to characterize and summarize the value of expert evidence. For a critique of such practices, see Refs. [5,21].

7 Note that this includes all the information available at the time a decision has to be taken. In the context of this paper, this includes, in particular, so-called prior information other than the specific scientific evidence received. See also [21] for a recent discussion, based on a real case, of the importance of priors in evaluating scientific evidence.

8 See also Kaye [18, p. 5] for related discussions of different understandings of Blackstone's widely quoted phrase “better that ten guilty persons escape, than that one innocent suffer” [9, at p. 352], which has become known as the “Blackstone ratio” [e.g., 20].
